# Methodological differences in fecal corticosterone metabolite extraction in pet rabbits: implications for welfare assessment

**DOI:** 10.3389/fvets.2026.1859281

**Published:** 2026-06-30

**Authors:** Martina Frühauf Kolářová, Katarína Kováčová, Michaela Součková, Miroslav Svoboda, Michal Zeman

**Affiliations:** 1 Department of Veterinary Sciences, Faculty of Agrobiology, Food and Natural Resources, Czech University of Life Sciences Prague, Prague, Czechia; 2 Department of Animal Physiology and Ethology, Faculty of Natural Sciences, Comenius University Bratislava, Bratislava, Slovakia; 3 Department of Ethology and Companion Animal Science, Faculty of Agrobiology, Food and Natural Resources, Czech University of Life Sciences Prague, Prague, Czechia

**Keywords:** extraction methodology, fecal corticosterone metabolites, HPA-axis, non-invasive assay, pet rabbits, stress

## Abstract

**Introduction:**

Valid quantification of fecal corticosterone metabolites (FCM) is a valuable non-invasive method for assessing hypothalamic-pituitary-adrenal (HPA) axis activity and stress responses in animals. The increasing emphasis on animal welfare highlights the need not only to expand knowledge but also to critically evaluate methodological approaches, particularly with regard to how individual procedural steps may influence results and contribute to variability or bias. Methodological inconsistencies between studies can significantly affect the accuracy and comparability of results. This study aimed to optimize the extraction procedure of corticosterone metabolites from feces of domestic rabbits (*Oryctolagus cuniculus domesticus*).

**Methods:**

We experimentally compared the effects of sample weight (50, 100, and 500 mg), extraction protocol (one-step vs. two-step), and dilution factor (10 × and 100 × ) using 80% methanol and commercial ELISA kit.

**Results:**

The optimal performance, in terms of extraction efficiency and reproducibility, was achieved using 100 mg of lyophilized and homogenized feces in a two-step methanol extraction with 3 + 2 mL. This configuration yielded a recovery rate of 51.55% and minimized matrix interference. Lower or higher sample weights resulted in technical artifacts or low extraction efficiency.

**Discussion:**

The optimized method provides a standardized, reproducible, and feasible approach for the robust non-invasive assessment of adrenocortical activity in rabbits and other small mammals. These results underscore the necessity of detailed methodological reporting to ensure data validity and comparability of results across studies, assuming the same immunoassay is used.

## 1 Introduction

The growing popularity of rabbits as companion animals has been accompanied by increasing attention to their welfare, particularly in the context of stress associated with human–animal interactions (1–5). However, existing research has predominantly focused on behavioral indicators, while physiological markers remain comparatively underexplored despite their importance for a comprehensive understanding of stress responses (6, 7). These approaches are particularly relevant for rabbits as a prey species, in which behavioral signs of stress responses may often remain subtle or hidden (8, 9).

Corticosterone, the main glucocorticoid in rabbits and some rodents (such as mice and rats), plays a crucial role in the control of energy metabolism, immune responses, and the stress response (10–15). While its short-term effects promote adaptive processes, chronically elevated levels of this hormone can lead to pathological consequences, such as the disruption of reproductive biology (16), impairment of cognitive function (17), and overall deterioration of health (18, 19). Traditional invasive methods of corticosterone measurement from blood require direct contact with the animal, which is a stressor that significantly affects hormone levels within minutes (20–25). Therefore, non-invasive techniques that minimize these negative effects are needed. They are particularly valuable in behavioral studies, welfare assessments, physiological monitoring, and the analysis of the effects of environmental changes (12, 26). Non-invasive approaches include the analysis of glucocorticoids across species from various biomaterials, e.g., feces (27, 28), fur (29, 30), feathers (31), shed snake skin (32), claws (33, 34), and earwax (35).

Glucocorticoids are metabolized in the liver, and their metabolites are excreted in urine or feces (36–38). The excretion of glucocorticoid metabolites in feces occurs with a species-specific time delay that approximately corresponds to the time of passage through the intestine (37, 39, 40). In rabbits, previous studies have repeatedly shown that fecal corticosterone metabolites (FCM) are a reliable biomarker of the hypothalamic-pituitary-adrenal (HPA) axis (13–15, 41–43). Fecal sample collection in rabbits is a non-invasive method that does not require manipulation or restraint of the animal, allows repeated sampling, and can be performed at any time of day without the need for specially trained personnel (14).

Over the past two decades, a number of studies using FCM measurements have been published (14, 44–46), in which the FCM extraction procedures varied significantly between laboratories. They differ in terms of sample weight, processing methodology, extraction duration, and whether or not the extract is evaporated. For example, studies using comparable sample-to-solvent ratios (approximately 1:10) but short extraction times (e.g. ~30 min of shaking) have reported markedly divergent FCM concentrations, ranging from tens of pg/g (44, 47) to tens of ng/g (45). Similarly, increasing extraction efficiency through modification of the solvent or protocol may result in substantially higher values, as demonstrated by Buijs et al. (48), who reported concentrations of 250–400 ng/g using methanol-based extraction with a comparable extraction time. An even more pronounced effect is observed when the extraction duration is extended; while Monclús et al. (14) reported values of approximately 70–90 ng/g following a 30-min extraction (shaking), Navarro-Castilla et al. (46), applying a similar approach but extending the extraction time to 16 h (shaking), obtained concentrations exceeding 700 ng/g. These findings indicate that, although the sample-to-solvent ratio represents an important parameter, it is the duration and intensity of extraction that play a decisive role in the release of metabolites from the fecal matrix, potentially leading to differences in measured concentrations of several orders of magnitude. Furthermore, insufficient documentation of methodological details with the potential to influence analytical outcomes limits the practical application of these procedures and poses a significant challenge. A precise description of these methodological details and the validation of the extraction processes are essential to increase the validity of the results obtained and their use. These factors include, for example, the specifics of the mechanical preparation of the sample, the timing of individual steps, the length of sample preparation, and the precise parameters of centrifugation (49, 50).

Commercial ELISA kits for measuring FCM offer a relatively simple and practical approach for non-invasive stress assessment (51). Nevertheless, despite the straightforward assay procedure, errors and variability in results may still occur, which can affect the reliability of the measurements (49). In addition, sample preparation and extraction require careful handling and appropriate laboratory practice; therefore, all procedures should be thoroughly documented and standardized to ensure valid and comparable data.

The aim of this study was therefore to analyze and clarify the key methodological steps for the extraction of corticosterone metabolites from domestic rabbit feces. The experimental process was designed to systematically compare extraction procedures reported in the published literature and to assess the influence of sample weight, extraction time and dilution rate on the resulting extraction efficiency, with particular emphasis on developing a non-invasive approach that preserves animal welfare.

## 2 Materials and methods

### 2.1 Animals and sample collection

Twelve clinically healthy, 6-month-old, unneutered female dwarf rabbits were included in the study. They were kept under the same hobby husbandry conditions at room temperature (19–21 °C), fed hay *ad libitum* and pelleted feed at the same regular intervals with free access to fresh water. Fecal samples were collected from each individual 3 times on independent collection days with 7–10 day intervals to cover the course of the entire month, including varying estrous activity. To minimize the influence of diurnal variability in glucocorticoids, all samples were collected around 9 a.m. after feeding. Samples were collected immediately after defecation, labeled, and frozen at −80 °C until analysis. Before the extraction procedure began, individual fecal samples from all 12 rabbits were mixed and pooled to create a single composite sample, which was subsequently used throughout the optimization experiment to minimize biological variability and enable direct comparison of individual processing steps.

### 2.2 Extraction procedure

**Figure 1:**
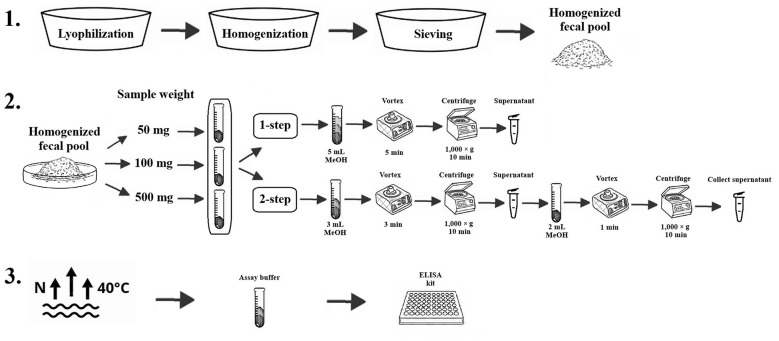
Schematic overview of the experimental procedure. The diagram illustrates the main methodological steps, including sample preparation and extraction, as well as the comparison of extraction approaches. 1. Lyophilization, homogenization, and sieving of pooled fecal material to obtain a dry, particle-free, and homogeneous sample. 2. Different initial samples weights (50, 100, 500 mg) were weighed, followed by one-step (5 mL MeOH), or two-step methanol extraction procedures (3 + 2 mL MeOH). 3. Evaporation of methanol under a gentle stream of nitrogen (N) at 40 °C, resuspension with assay buffer and ELISA analysis.

The experimental design followed a full-factorial approach, in which the effect of three factors: three sample weights (50, 100, and 500 mg), washing protocols (one-step and two-step washing), and two dilution factors (10 × and 100 ×) were evaluated simultaneously within a single experimental run. In addition, three spiking conditions (unspiked control, +500 pg CORT, and +1,000 pg CORT) were applied across all experimental combinations to assess recovery. This resulted in 36 unique experimental conditions (3 × 2 × 2 × 3), each performed in duplicate, yielding a total of 72 measurements. Six measurements were not included in the final dataset, resulting in 66 measurements available for analysis. The entire procedure is illustrated in Figure 1.

All methodological factors were evaluated using aliquots derived from the same homogenized fecal pool to enable direct comparison of methodological variables under identical experimental conditions and minimized inter-assay variability associated with separate experimental runs. Recovery was assessed by spiking aliquots with known amounts of corticosterone prior to extraction, while the control aliquots remained unspiked. These aliquots were processed simultaneously but represent analytically distinct conditions. Spiked aliquots received either 500 or 1,000 pg of corticosterone prior to extraction.

The extraction procedure was based on the basic steps published in Touma et al. (52), Palme (50), and Monclús et al. (14). The preparation of samples for the FCM assay included lyophilization (53), homogenization and sieving. The mixed fecal pool sample was lyophilized using a lyophilizer (Lyovac GT 2 Steris, SRK Systemtechnik GmbH, Germany) to remove water from the sample (dried for 24 h at −18 °C). Subsequently, samples were ground in an IKA grinder (IKA A11 basic, IKA-Werke GMBH & CO.KG, Staufen, Germany). The sample was then sieved through 2 stainless steel sieves with different mesh sizes (1.5 mm and 1 mm mesh diameter) to remove large particles, especially pieces of hay. The sample was thoroughly mixed to ensure homogeneity. From this homogenized sample pool, individual samples of 50, 100, and 500 mg were weighed and then subjected to the extraction procedure.

Each sample was extracted with 5 mL of 80% methanol in two different ways: 1-step and 2-step extraction. Half of the samples were extracted by a one-step process—samples were extracted once with 5 mL of 80% methanol, centrifuged (1,000 × g for 10 min), and the supernatant was transferred into clean tubes. To the other half of the samples (2-step process: 3 + 2 mL, in total 5 mL), 3 mL of 80% methanol was added, mixed thoroughly, and centrifuged for 10 min. The supernatant was transferred to a clean tube. Then, 2 mL of 80% methanol was added to the remaining pellet, mixed, and centrifuged again. The second supernatant was combined with the first to obtain the final extract.

All samples were subsequently vortexed at high speed (5 min for 1-step, 3 + 1 min for 2-step extraction) and centrifuged at 1,000 × g for 10 min, and the supernatant was transferred to clean tubes. Due to potential contamination, the samples were centrifuged again for 10 min. Methanol was then evaporated under a gentle stream of nitrogen at 40 °C (Reacti-Therm III TS-18824, Thermo Fisher Scientific, Waltham, MA, USA) according to the ELISA kit manufacturer's recommendations. It is important for the samples not to turn brown. Dry samples were resuspended in 0.5 mL (for 50 and 100 mg weight) or 1 mL (for 500 mg) of assay buffer. The reconstituted samples were vortexed for 30 s and left to stand briefly, vortexed again for 30 s, and centrifuged at 1,000 × g for 5 min at 4 °C. The supernatant was transferred into microtubes. In the subsequent step, samples were diluted in assay buffer at either 10 × or 100 × , and each dilution was assayed in duplicate on a single plate.

### 2.3 ELISA

A commercially available ELISA Corticosterone kit (Cayman Corticosterone, Cayman Chemical, Ann Arbor, MI, USA), a competitive test developed for measuring corticosterone in serum, feces, and other matrices, was used to determine corticosterone and its metabolites in feces. The test range was 8.2–5,000 pg/mL, and the sensitivity (80% B/B0) was approximately 30 pg/mL. The plate was read on an absorbance reader (BioTek Epoch 2 Microplate Spectrophotometer, Agilent, Santa Clara, CA, USA) after 90–120 min at a wavelength of 412 nm, when the absorbance in well B0 reached ≥ 0.3 A.U. The intensity of the color, determined spectrophotometrically, is inversely proportional to the amount of free corticosterone present in the well during incubation. The analysis was performed in duplicate of diluted samples in a single plate. The intra-assay coefficient of variation for duplicates was 4.54%.

The web application provided by the ELISA kit manufacturer (www.caymanchem.com/analysis/elisa) was used to verify the calculations, a standard curve was generated from the ELISA results, and FCM concentrations were subsequently determined in pg/mL, with conversion to pg/mg of homogenized fecal material performed using the following formula.


$$\begin{array}{l} c_{1}=\frac{c_{0}\;\times d\;\times r}{w}\; \end{array}$$

Where:

**c**_**1**_ is the resulting FCM/corticosterone concentration (pg/mg).

**c**_**0**_ is the FCM/corticosterone concentration (pg/mL) obtained from the standard curve.

**d** is the sample dilution performed immediately prior to analysis (10 × or 100 × ).

**r** is the volume (mL) of ELISA buffer used for sample reconstitution before analysis.

**w** is the weight (mg) of the sample after lyophilization, grinding, and sieving of the feces.

### 2.4 Statistics

Statistical analysis was performed and graphs were generated using GraphPad Prism 9 (GraphPad Software, Boston, MA, USA). Data normality was assessed using the Shapiro-Wilk test. FCM concentrations between the two extraction approaches were compared using a Mann-Whitney test. Differences in FCM concentrations between batches were evaluated using the one-way ANOVA test followed by Tukey's *post hoc* test. Recovery was determined by comparing the measured FCM concentration in spiked and unspiked samples of different sample weights to assess the effect of sample mass on extraction efficiency and matrix interference.

## 3 Results

### 3.1 Solvent selection and sample solution

We used 80% methanol for the extraction and subsequently tested the assay performance using two dilution factors (10- and 100- fold). The averaged corticosterone concentrations calculated directly from the standard curve, prior to correction for dilution, were 1,064.4 ± 87.7 pg/mL (*n* = 34) and 86.9 ± 5 pg/mL (*n* = 32) for samples diluted 10- and 100-fold, respectively. After correcting the raw values for dilution and normalization to the dry weight of the feces, the mean concentration of FCM in samples was 45.2 ± 2.5 pg/mg and 40.2 ± 2.8 pg/mg for 10- and 100-fold dilution, respectively. Although mean concentration was 11% lower in the 100-fold dilution, the effect of the dilution factor was not statistically significant (*p* = *0.22*).

### 3.2 Optimization of sample quantity for analysis

**Figure 2:**
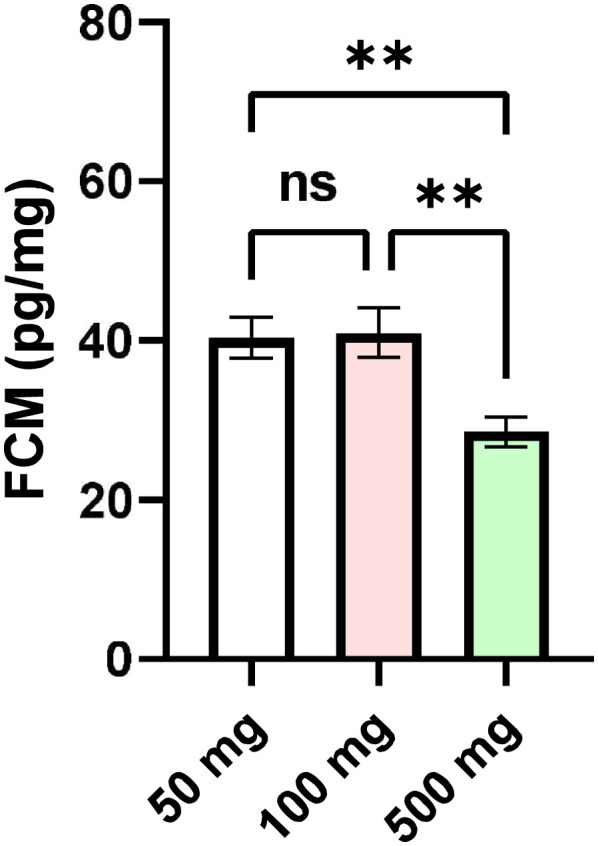
Concentration of fecal corticosterone metabolite. Fecal corticosterone metabolite (FCM; pg/mg) according to different sample weight–50, 100, or 500 mg (*n* = 7 per 50 and 500 mg, *n* = 6 per 100 mg weight). Data are presented as mean ± SEM; **p < 0.01; ns, non-significant.

Three different initial weights were tested to determine the optimal sample weight: 50, 100, and 500 mg. Indeed, when we compared FCM concentrations across sample weights, we found significant differences (*F* = 7.93, *p* = *0.004*). *Post hoc* analysis showed that FCM concentration was significantly lower in 500 mg sample weight compared to 100 mg sample weight (*p* = *0.008*) and compared to 50 mg (*p* = *0.009*). No significant differences in FCM concentration were observed between 50 mg and 100 mg (*p* = *0.98*, Figure 2).

**Figure 3:**
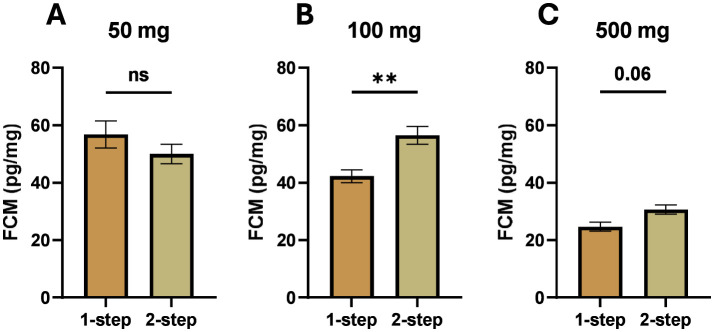
Concentration of fecal corticosterone metabolite. Fecal corticosterone metabolite (FCM; pg/mg) according to sample weight and methanol extraction process: 1-step—single extraction with – 5 mL; 2-step —repeated extraction with 3 + 2 mL; **(A)** 50 mg (*n* = 10 and 12), **(B)** 100 mg (*n* = 12 and 9), and **(C)** 500 mg (*n* = 11 and 12); Data are presented as mean ± SEM; **p < 0.01; ns, non-significant.

Next, we examined differences in FCM concentration between one and two-step methanol extraction. FCM concentrations were higher after the two-step extraction process for the 100 mg samples (*p* = *0.003*, Figure 3B). FCM concentration did not differ due to the extraction process in the 50 mg samples (*p* = *0.41*, Figure 3A). There was a tendency to a higher yield after two-step extraction for the 500 mg samples (*p* = *0.06*, Figure 3C), but the extraction efficiency was unacceptably low when extracting 500 mg of feces.

Our results show that using 50 or 100 mg of the dried fecal sample provides a higher and more efficient FCM yield than using 500 mg. A 5 mL volume of methanol was insufficient for the 500 mg sample in both the 1-step and 2-step extraction procedures.

**Figure 4:**
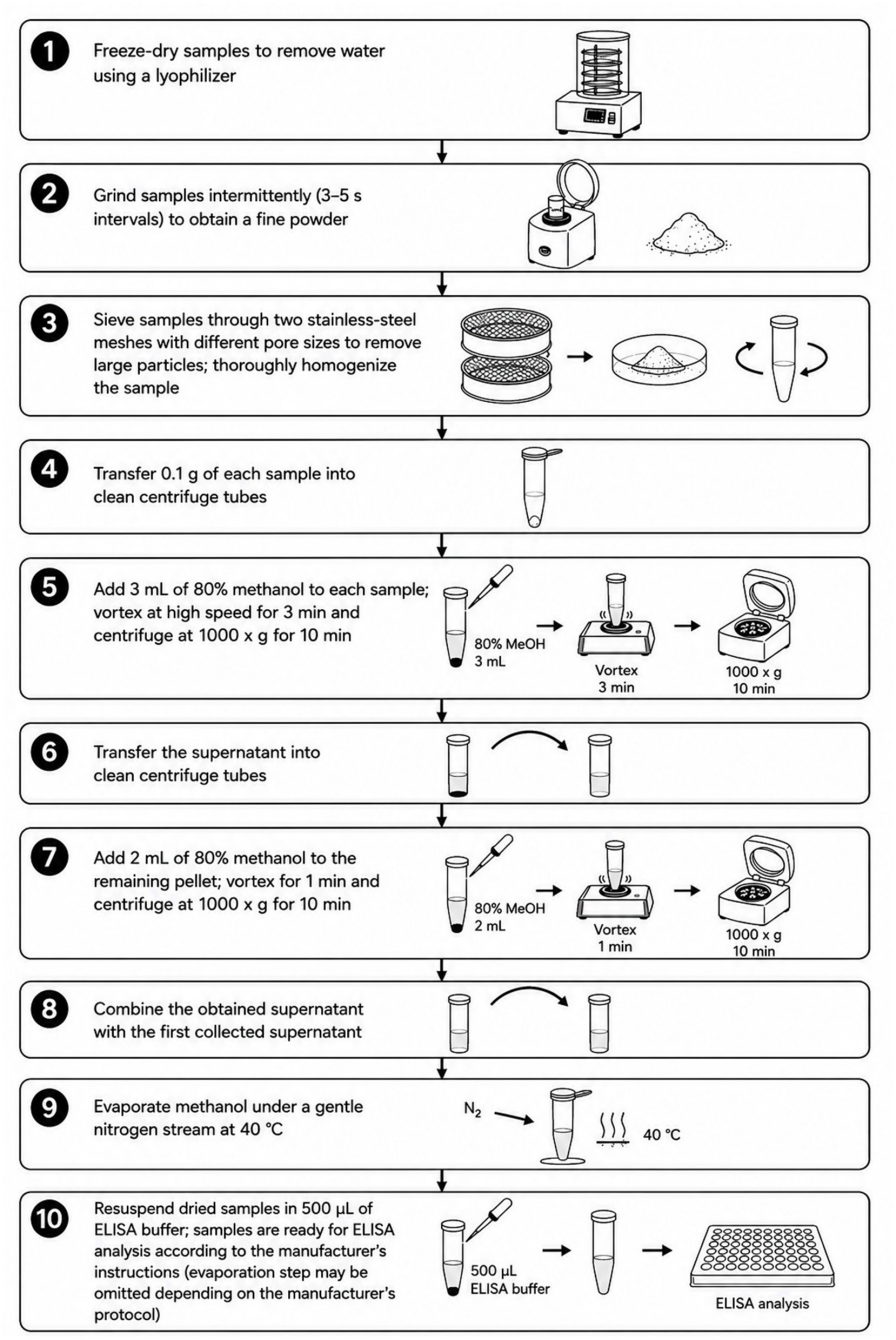
Workflow for extraction of fecal corticosterone metabolites (FCM) from rabbit fecal samples for subsequent ELISA analysis. Schematic overview of the sample preparation and extraction protocol. Fecal samples were first freeze-dried to remove moisture (1) and subsequently ground to a fine powder (2). The homogenized material was sieved through stainless-steel meshes to ensure uniform particle size and sample consistency (3). Aliquots (0.1 g) of the processed samples were transferred into centrifuge tubes (4) and extracted with 80% methanol: initially 3 mL with vortex mixing (3 min) followed by centrifugation (1,000 × g, 10 min) (5). The supernatant was collected (6), and a second extraction of the remaining pellet was performed using 2 mL of 80% methanol with shorter vortexing (1 min) and the same centrifugation conditions (7). Supernatants from both extraction steps were combined (8), and methanol was evaporated under a gentle nitrogen stream at 40 °C (9). The dried extracts were reconstituted in 500 μL of ELISA buffer and subsequently used for corticosterone metabolite quantification according to the manufacturer's instructions (10).

The recommended procedure for the 100 mg sample, illustrated in Figure 4, involves lyophilization, homogenization, and sieving of fecal material, followed by a two-step extraction with 80% methanol (3 + 2 mL) with vortexing and centrifugation, combination of supernatants, evaporation under nitrogen at 40 °C, and reconstitution of the dried extract in assay buffer prior to ELISA analysis.

### 3.3 Recovery experiments

Recovery experiments are used to validate the precision of immunoassays by estimating the recovery of known amounts of corticosterone added to samples prior to the extraction process. Therefore, FCM content was measured in samples without added corticosterone and in samples spiked with 500 pg and 1,000 pg of exogenous corticosterone. FCM concentration varied depending on sample weight (Table 1).

**Table 1:** Average FCM concentrations (pg/mg) in dried fecal samples of different weights with 0 (control), 500, or 1,000 pg of exogenous corticosterone.

Added corticosterone	50 mg (pg/mg)	100 mg (pg/mg)	500 mg (pg/mg)
Control	40.36 ± 2.6 (*n* = 7)	40.98 ± 3.1 (*n* = 6)	28.57 ± 1.9 (*n* = 7)
500 pg	54.59 ± 4.9 (*n* = 8)	46.90 ± 4.5 (*n* = 7)	27.43 ± 2.3 (*n* = 8)
1,000 pg	64.04 ± 1.6 (*n* = 7)	55.16 ± 3.1 (*n* = 8)	27.59 ± 2.5 (*n* = 8)

Values are presented as mean values ± SEM, with the number of replicates indicated in parentheses (n).

Three different sample weights (50, 100, and 500 mg) of fecal samples were tested to assess extraction efficiency and recovery. For the 50 mg and 100 mg samples, FCM concentration increased with the amount of added corticosterone, indicating more effective extraction and detection. The 500 mg samples showed little to no change in FCM concentration regardless of the amount of added corticosterone.

Recovery calculations were performed for each sample weight. Samples of 50 mg exhibited recoveries of 60.41% and 45.62% for the additions of 500 pg and 1,000 pg of corticosterone, respectively. Samples weighing 100 mg exhibited recoveries of 51.55% and 39.13% for 500 pg and 1,000 pg additions, respectively.

## 4 Discussion

The estimation of glucocorticoid metabolite concentrations in feces (FCM) is a promising non-invasive tool for monitoring hormonal changes and the body's physiological response to stress, enabling the long-term monitoring of stress responses, disease progression or therapeutic interventions. However, different methodological details during extraction or their insufficient description in publications may lead to incomparable results even when using the same immunoassay. Consequently, the results may not reflect only increased adrenocortical activity, but rather methodological variability related to the extraction procedure.

Because extraction is a critical step in FCM analysis, we evaluated three factors affecting extraction efficiency: different sample weights, two extraction methods, and dilution. Our results indicate that 100 mg of lyophilized and homogenized feces represented the most suitable approach overall. Although it did not provide the highest recovery, it achieved a favorable balance between recovery, matrix interference, assay reliability, and reproducibility in both one- and two-step extraction procedures. In contrast, 500 mg samples showed very low recovery for both corticosterone concentrations tested, suggesting that larger masses contain more matrix components or rigid structures that impair extraction or analyte detection. Consequently, 100 mg samples yielded the highest FCM concentration with minimal unwanted cross-reactivity and appear optimal for routine measurements of glucocorticoids in feces via this non-invasive assay.

The relatively low recovery values observed in this study likely reflect both methodological (careful preparation of samples prior to extraction) and biological factors rather than extraction inefficiency alone. One important aspect is the inherent mismatch between the spiked analyte (corticosterone) and the compounds measured by the assay, which consist of a heterogeneous mixture of fecal corticosterone metabolites. Differences in antibody cross-reactivity may therefore reduce the apparent recovery of exogenous corticosterone compared to endogenous metabolites. In addition, fecal matrices contain pigments, lipids, and microbial metabolites that can interfere with hormone extraction or antibody binding in immunoassays, thereby reducing the analyte detectability despite adequate extraction (54). It should also be noted that immunoassays applied to complex biological matrices often exhibit deviations from linearity in spike-and-recovery experiments, and lower recovery values in fecal hormone analyses have been reported in the literature (38, 49). Therefore, recovery values should be interpreted cautiously as they reflect both analytical and matrix-related influences. Generally, an extraction method with lower recovery but low variation is preferable to one with higher recovery but high variability. Nevertheless, further optimization of extraction conditions (e.g., extraction duration or solvent volume) may help to improve recovery in future studies.

To minimize individual variability and focus on methodological effects, a pooled composite sample was prepared from fecal collections repeatedly obtained from 12 female pet rabbits to approximate the mean FCM concentration (9). Pooling reduced inter-individual variability and the influence of outliers, providing a more representative matrix and improving reproducibility and consistency during method optimization.

Most published FCM studies use methanol (14, 38, 46, 48) or ethanol (44, 45, 47), typically at concentrations of 80% or 90%. Only a small number of studies used other solvents, such as water or buffer, dichloromethane, or diethyl ether. High-percentage aqueous methanol (typically 80%) is generally recommended for FCM extraction from mammalian feces (49, 55), and the same concentration was used in our study in accordance with the kit manufacturer's recommendations.

Another important consideration is the amount of sample used for extraction. We compared sample weights of 50, 100, and 500 mg. Although 50 mg corresponds to the manufacturer's recommendation and yielded results comparable to those obtained with 100 mg, it was less suitable for routine laboratory use because electrostatic charging and adhesion of fecal particles to tube walls increased the risk of weighing errors. Moreover, this lower sample weight may lead to overestimation of measured FCM concentrations, with measured values approaching the lower sensitivity limit of the standard curve under basal unstressed conditions. Nevertheless, a 50 mg sample may be useful when the amount of available fecal material is insufficient. In contrast, 500 mg samples exhibited low extraction efficiency and stronger matrix interference. Individual samples, irrespective of their mass, exhibited varying intensities of yellow-brown coloration, with some appearing distinctly darker. The extraction of 500 mg of feces using 5 mL of methanol resulted in a colored extract, low extraction efficiency, and very low measured final concentrations. The above-mentioned complications could explain the lower FCM levels. It is therefore necessary to consider the volume of methanol used, as 5 mL may not be sufficient for complete extraction of larger samples. Therefore, a higher volume of methanol should be considered in future studies if sample weights greater than 100 mg of feces are to be used. We used 5 mL of methanol uniformly in our study to ensure the simplicity and uniformity of the sample preparation. Some published studies used 200 mg samples (14, 44), which can sometimes be difficult to obtain. Because 100 mg provided adequate recovery while remaining technically feasible, we recommend this sample weight, particularly for small mammals, where sample quantities may be insufficient. Importantly, many studies fail to specify whether reported sample weights refer to fresh or lyophilized feces. Our results emphasize that sample weight after lyophilization and homogenization should always be clearly reported.

As part of method validation, we also compared one- and two-step extraction procedures. A significant difference was observed for 100 mg samples, supporting the two-step procedure as the preferred approach. Although no significant difference was detected for the 500 mg samples, the two-step extraction showed a tendency toward higher extraction efficiency. These results indicate that, although repeated extraction using 3 and 2 mL of methanol is more time-consuming, it may improve analyte recovery, particularly for larger sample masses, where the increase in extraction efficiency may justify the additional step while allowing more efficient use of the extraction solvent. For lower-mass samples, a one-step extraction may be sufficient, especially as a time-saving alternative.

Sample dilution was performed according to the ELISA kit manufacturer's recommendations (10 × and 100 × ). Commercial ELISA tests are a convenient solution for laboratory analysis due to their ease of use and availability, despite their relatively high price (49). Our uncorrected data suggest that the 100-fold dilution better fits the standard curve range, since the values were in the upper, more sensitive part of the standard curve. This higher dilution can be more advantageous when samples from stressed animals are measured and higher FCM values are expected. Higher dilution also reduces the non-specific matrix interference in the ELISA assay. However, it is necessary to consider that due to differences in the affinity of antibodies to different glucocorticoid metabolites, the results may vary considerably between individual tests. FCM measurements therefore depend largely on the methodology used, and results cannot be easily compared between studies unless identical methodologies are used (28, 56). Since native glucocorticoids are rapidly metabolized in the body, only their stable metabolites (28, 50), which are biologically inactive, should be assayed (57).

In addition to the above-mentioned variables, specific sources of error and variability in the measurement of glucocorticoid metabolites in feces include dietary factors and storage time. For welfare assessment based on FCM, it is therefore essential to standardize all potential sources of variation as much as possible while accounting for inherent inter-individual variability. Consequently, longitudinal monitoring of the same individuals over time is generally preferable to direct comparisons between individuals. In line with this approach, all samples should be processed using a standardized protocol.

In our laboratory study, these conditions were strictly controlled: all animals were maintained under a uniform feeding regime throughout the experiment, samples were collected at consistent times and intervals, and immediately frozen after collection.

Moreover, FCM concentrations can be affected by several biological variables that must be carefully considered when interpreting the data (12, 27, 50, 56, 58). Elevated FCM levels may therefore reflect not only changes in welfare status but also confounding biological factors or methodological differences (59, 60). Among the biological factors, age and sex should be considered. Several studies confirmed differences in FCM levels between males and females (59–61) and between ages or developmental stages (62, 63). To minimize these sources of variability, we included only females of a similar age in our study.

## 5 Conclusions

This study focused on optimizing the methodology for the extraction and determination of corticosterone metabolites from feces of domestic rabbits with the aim of optimizing the accuracy, precision, and reproducibility of non-invasive stress marker measurements. An experimental comparison of different sample weights, extraction methods, and dilutions showed that for optimal measurement—defined in terms of extraction efficiency and the optimum amount of feces with respect to the assay requirements and the size of the animals—the use of 100 mg of lyophilized and homogenized feces in conjunction with a two-step extraction using 80% methanol is recommended. This approach provided the most reproducible results and can be recommended as a reliable and effective system for non-invasive stress assessment in domestic rabbits and other small mammal species. These results also highlight the key role of careful standardization of lyophilization, homogenization, and consistent extraction procedures to achieve reliable and comparable data. These methodological details should be consistently monitored and reported in future studies to obtain valid results that are comparable across laboratories. This issue must be carefully considered when applying HPA axis monitoring in ethological studies and in the assessment of welfare in pet rabbits, where non-invasive stress evaluation is essential for the accurate interpretation of behavioral data and for ensuring high standards of animal welfare. Thus, this methodology has the potential to increase the validity of the non-invasive monitoring of glucocorticoid metabolites in studies dealing with stress, welfare, ecophysiology, ethology, animal protection, and biomedicine. However, further studies are needed to validate our recommendations when the assay is used to compare stress levels in different rabbits.

## Data Availability

The original contributions presented in the study are included in the article/supplementary material, further inquiries can be directed to the corresponding author.
